# Molecular Research and Treatment of Breast Cancer

**DOI:** 10.3390/ijms23179617

**Published:** 2022-08-25

**Authors:** Anna Kawiak

**Affiliations:** Intercollegiate Faculty of Biotechnology, University of Gdansk, Abrahama 58, 80-307 Gdansk, Poland; anna.kawiak@biotech.ug.edu.pl

Breast cancer is the leading cause of cancer-related deaths in the female population [1]. The complexity of the molecular mechanisms that regulate tumor initiation and progression determines the heterogeneity of breast cancer. At the molecular level, this diversity poses a challenge in the selection of treatment options and disease prognosis [2]. Advances in molecular research have allowed for a greater understanding of the cellular pathways that govern breast tumor development, facilitating progress in identifying diagnostic markers and the development of novel therapeutic strategies, some of which are presented in this Special Issue.

The diversity of therapy outcomes for breast cancer patients with similar prognostic variables calls for further advances in the identification of novel prognostic markers for the improvement of clinical prognosis [3]. Transcription factors are considered important markers of prognostic and predictive value; since they are drivers of tumor development and progression, this makes them valuable prognostic and therapeutic targets [4]. Ogura et al. [5] identified the transcription factor octamer transcription factor 1 (OCT1) as a novel prognostic factor for estrogen receptor-positive breast cancer. OCT1 regulates the expression of genes involved in processes such as cell proliferation and metastasis. Positive OCT1 immunoreactivity (IR) was found to be a poor prognostic factor in ER-positive breast cancer. The identified OCT1 target gene *NCAPH* positively correlated with OCT1 IR and was also found to be associated with poor prognosis. OCT1 and *NCAPH* promoted the proliferation of breast cancer cells and long-term estrogen-deprived (LTED) cells, suggesting their role in estrogen resistance and pointing to not only their prognostic, but also their therapeutic value in ER-positive breast cancer [5].

Another molecular target in breast cancer, functioning as a cotranscription factor, is nuclear EGFR (nEGFR). EGFR signaling functions at the plasma membrane and regulates genes in the nucleus involved in tumor progression. nEGFR mediates triple-negative breast cancer (TNBC) resistance to anti-EGFR agents, such as cetuximab [6]. The antimalarial drug primaquine was found to inhibit the nuclear translocation of EGFR in TNBC by inducing endocytosis-mediated EGFR degradation. nEGFR interacted with the DNA-binding transcription factor STAT3, activating the transcription of genes involved in cell cycle progression and apoptosis in the nucleus. Primaquine inhibited Stat3/nEGFR interactions and induced apoptosis through the downregulation of c-Myc, providing a therapeutic strategy for TNBC by targeting nEGFR signaling [7].

An important aspect of improving the efficacy of drugs currently used in breast cancer is delineating the mechanisms responsible for drug resistance. Drug resistance is a multifactorial and multistep process, and advances in molecular research have identified multiple involved mechanisms, including the activity of drug-metabolizing enzymes, drug efflux, and glutathione detoxification systems. Changes in drug targets, DNA damage repair mechanisms, and the overexpression of apoptosis-related factors also contribute to the development of drug resistance. The response of cancer cells to drugs is not solely associated with intrinsic mechanisms, but is also dependent on signals obtained from the tumor microenvironment, playing an important role in tumor progression and therapy response, mediating drug resistance. The epithelial-to-mesenchymal transition (EMT), associated with the enhanced migratory and invasive potential of breast cancer cells and their increased stemness, also contributes to drug resistance [8]. The epithelial–mesenchymal transition (EMT) and cancer stem cell (CSC) properties were acquired in doxorubicin-resistant breast cancer cells. Doxorubicin-based chemotherapy is one of the most frequently used systemic triple-negative breast cancer (TNBC) treatments. The upregulation of EGFR signaling was suggested to be involved in doxorubicin-acquired resistance. Doxorubicin-induced EMT and CSC properties of resistant cells were transferred from doxorubicin-resistant cells to doxorubicin-sensitive cells through autocrine signaling [9].

Novel drug delivery systems aiming to improve drug efficacy and minimize systemic toxicity are currently being explored, including ligand-mediated drug delivery methods as well as stimuli-responsive drug delivery systems. Esawi et al. [10] presented an aptamer chimera that targets doxorubicin delivery into breast cancer cells. This chimera consisted of an AS1411 DNA aptamer that recognizes nucleolin receptors, overexpressed in breast cancer, and an ATP aptamer loaded with doxorubicin for stimuli-triggering release. The AS1411–ATP aptamer chimera was demonstrated to release doxorubicin into the nucleus of cancer cells in response to ATP, inhibiting cell growth. The selective delivery of doxorubicin could decrease its off-target cytotoxicity and increase the efficacy of this agent [10].

Apart from novel drug delivery systems, novel targeted therapeutics are sought after. Among targeted drugs used in the treatment of breast cancer, poly-(ADP)-ribose polymerase (PARP) inhibitors (PARPis) and cyclin-dependent kinase 4/6 (CDK4/6) inhibitors (CDK4/6is) were approved in the clinical setting [11]. Combination treatment with PARPi and CDK4/6i showed improved efficacy in breast cancers, regardless of the homologous recombination (HR) proficiency [12]. Furthermore, the development of compounds comprising dual targeting activities exceeded the efficacy of mono- and combination therapy with PARP and CDK4/6 inhibitors. One such compound, ZC-22, inhibited breast cancer cell proliferation through DNA damage induction and cell cycle arrest. ZC-22 exhibited higher activity than the combination of PARPi olaparib and CDK4/6i abemaciclib and sensitized breast cancer cells to the activity of cisplatin, indicating its potential application in mono- and combination therapy [13].

Another important strategy in drug design is the development of agents capable of inhibiting the metastasis of cancer cells and the evaluation of their activity toward cell migration and invasion. The research of Meyer et al. [14] showed that drugs could elicit a diverse response on the migration of tumor cells, depending on cell–cell and cell–extracellular matrix interactions. Breast cancer cells treated with staurosporine displayed different migratory properties, which were context-dependent, subject to the presence of single or collective cells, and on growth substratum. Thus, the influence of a drug on the migration of tumor cells can be dependent not only on the cell line type, but also on the particular cellular context, which should be examined in order to fully elucidate the impact profile of a drug [14].

In summary, advances in the elucidation of molecular mechanisms regulating breast cancer progression and response to treatment have fueled the identification of novel molecular targets and treatment strategies. The articles within this Special Issue cover a wide range of research topics related to these advances, from the identification of prognostic markers to the development of targeted therapies. The findings presented in this Special Issue contribute to improving our understanding of the complexity of molecular mechanisms associated with breast cancer, and highlight the importance of elucidating these mechanisms.
